# Time experience in patients with schizophrenia and affective disorders

**DOI:** 10.1192/j.eurpsy.2022.2

**Published:** 2022-01-31

**Authors:** Paraskevi Mavrogiorgou, Theresa Thomaßen, Frederike Pott, Vera Flasbeck, Holmer Steinfath, Georg Juckel

**Affiliations:** 1 Department of Psychiatry, LWL University Hospital, Ruhr University Bochum, Bochum, Germany; 2 Department of Philosophy, Georg-August-University Göttingen, Göttingen, Germany

**Keywords:** Depression, schizophrenia, time experience, time feeling, time perception

## Abstract

**Background:**

The experience of time, or the temporal order of external and internal events, is essential for humans. In psychiatric disorders such as depression and schizophrenia, impairment of time processing has been discussed for a long time.

**Aims:**

In this explorative pilot study, therefore, the subjective time feeling as well as objective time perception were determined in patients with depression and schizophrenia, along with possible neurobiological correlates.

**Methods:**

Depressed (*n* = 34; 32.4 ± 9.8 years; 21 men) and schizophrenic patients (*n* = 31; 35.1 ± 10.7 years; 22 men) and healthy subjects (*n* = 33; 32.8 ± 14.3 years; 16 men) were tested using time feeling questionnaires, time perception tasks and critical flicker-fusion frequency (CFF) and loudness dependence of auditory evoked potentials (LDAEP) to determine serotonergic neurotransmission.

**Results:**

There were significant differences between the three groups regarding time feeling and also in time perception tasks (estimation of given time duration) and CFF (the “DOWN” condition). Regarding the LDAEP, patients with schizophrenia showed a significant negative correlation to time experience in terms of a pathologically increased serotonergic neurotransmission with disturbed time feeling.

**Conclusions:**

Impairment of time experience seems to play an important role in depression and schizophrenia, both subjectively and objectively, and novel neurobiological correlates have been uncovered. It is suggested, therefore, that alteration of experience of time should be increasingly included in the current psychopathological findings.

## Introduction

Everything is in time. All internal and external events are organized in time for humans and are experienced and arranged by them as before/after or in the past, present, or future. Changes in this time experience have been a recurring theme in the psychiatric literature [1]. In older, more phenomenological works, subjective changes in the sense of an altered feeling of time (“time becomes slow”) were reported; in studies of recent years, empirical research approaches regarding restrictions of so-called objective time perception have dominated [2,3]. Besides that, it was repeatedly found that basal temporal processing, is only changed in organic brain illnesses and objective perception of time is changed more in patients with schizophrenia than in those with depression, whereas the subjective feeling of time is disturbed in many psychiatric illnesses [1].

Depression is one of the most common psychiatric disorders and its incidence is steadily increasing [4], although the pathophysiology of this disorder is not yet fully understood. A multifactorial process of genetic predisposition, traumatic experiences, chronobiological changes, and current psychosocial stress is assumed as the cause. These factors, and their interaction, can lead to an impairment of neurotransmitter systems such as the serotonin system, which could explain the depressive symptoms [5]. A serotonin deficiency postulated in depressive patients affects the entire central nervous system, but especially the limbic system, via the modulating projection pathways of the serotonergically innervated raphe nuclei. From this, a serotonergic influence on emotional processes, as well as on the sleep–wake rhythm and time experience, is derived in general and for depression and schizophrenia specifically [6].

The suspicion that an altered experience of time could be a decisive influencing factor in depression first arose at the beginning of the 20th century. The impetus for this came from von Gebsattel [7], who published a phenomenological case report in his article “Time-related obsessive thinking in melancholia.” As the core of their depression, von Gebsattel thematized an altered time feeling. Von Gebsattel impressively described the patient’s “time-related compulsion to register” [7, p. 278], through which she experienced an increasing senselessness of her own existence and developed depressive symptoms, such as a loss of interest and lack of drive. Based on this individual definition of time as the basis of the depressive symptoms, which today would perhaps rather be described as the phenomenon of “compulsive slowness” [1], von Gebsattel generalized the subjectively altered experience of time, in the sense of the presence of the past and concentration on the passing of time, as a phenomenon underlying depression. Against the background of this early individual case study, the assumption of a disturbed experience of time in depressive patients has been taken up many times [1].

It is now well documented that during a depressive episode many patients have the subjective impression of standstill and the feeling that time is passing very slowly [8]: “They experience a past that is becoming overpowering and a small insignificant present.” At the level of objectively measurable time perception, an attempt was made to find an empirically provable correlate to the subjective impressions of the sufferers. In 2015, Thönes and Oberfeld published a meta-analysis on the experience of time in depression and found in many studies that depressive patients would report their time feeling as being much slowed. However, in the meta-analytical examination of 16 empirical studies, no significant influence of depression and measured depressiveness (psychopathologically and psychometrically) on four dimensions of objective time perception (discrimination, reproduction, production, and estimation) could be found. The authors assumed the model of an “inner clock” for the phenomenon of slowed time perception especially for duration evaluation. This consists of a pacemaker that sends impulses and a counter that processes these impulses. As the speed of the temporal impulses emitted by the pacemaker differs, it should be possible to detect objective differences in the processing of various time perception tasks compared to the healthy control group. However, only differences regarding time feeling, but not time perception could be determined across all previous studies [2].

Change in the experience of time has also been described for patients with schizophrenia. Already at the beginning of the 20th century, Franz Fischer in 1929 [9] reported in his article “Zeitstruktur und Schizophrenie” (Time Structure and Schizophrenia) case descriptions of an objectification and a “moving away” of time in the context of this disorder. Fuchs [10] then later spoke of a fragmentation of the stream of consciousness in schizophrenic patients. Kupke [11], in his book “Der Begriff Zeit in der Psychopathologie” (The Concept Time in Psychopathology), even cited an altered time experience as an explanatory approach for the development of ego disturbances: “They notice that they are no longer the master of their very own inner experience of time. So, it seems logical to think that there is an alien spirit inside them that dominates them” [p. 54].

In addition to individual case descriptions on the topic of time experience in schizophrenia, there are also numerous empirical studies on the topic of time perception. These were summarized in 2017 by Thönes and Oberfeld as part of a meta-analysis. More than 68 publications from 1956 to 2014 were analyzed, examining time perception (i.e., the assessment of a time interval and the perception of the temporal sequence of two stimuli). The main finding of the meta-analysis is that patients with schizophrenia are particularly impaired in the precision of time perception, since their values showed a broader range than that of healthy control subjects. However, in regard to the accuracy with which the time intervals were estimated, no significant difference could be found compared to the control group. According to the authors, it is possible that the effects would be greater without medication. As a theoretical explanatory model for the measured effects of the altered time perception, reference is again made to the model of the “inner clock” by Treismann [12] which focus especially on duration evaluation: “In patients with schizophrenia, this inner clock is accelerated so that more impulses are registered per time interval, which leads to a temporally prolonged perception of the interval. This acceleration thus manifests itself in a greater variability and overestimation of the duration.”

Previous studies of time experience in the sense of subjective time feeling and objective time perception are subject to several difficulties. The pure observation of time experience as a feeling of time is difficult to access from an external psychological point of view and can therefore hardly be objectified in a scientifically valid way. When measuring time perception, different individual memory and cognitive processing could be activated within the framework of methodological heterogeneity and different tasks. This is particularly important in the tasks for the discrimination of time intervals, because sensory processing must be assumed in the differentiation of intervals in the millisecond range, whereas for intervals of one or more seconds cognitive factors such as attention and memory become increasingly important. So far, it has remained unclear how the subjective time feeling can best be scientifically examined as a psychopathological abnormality without recourse to the phenomenological individual case level.

In order to address such points of criticism, the additional investigation of neurobiological parameters as possible objective correlates, namely measurement of the loudness dependence of acoustic evoked potentials (LDAEP) and the critical flicker-fusion frequency (CFF), should take place within the present study on time feeling and time perception. In order to be able to determine the activity of the synaptically released serotonin in the central nervous system, measurement of the LDAEP increasingly has proved to be a valid indicator for this in recent years [5], which is supported by numerous studies on depression and schizophrenia to date [13]. Interestingly, the serotonergic and the dopaminergic system, which was more in the focus concerning temporal processing so far, is closely intertangled with the lead by the oldest neurotransmitter [14]. Besides serotonin and dopamine, there are definitively several further neurotransmitters being involved in such a complex issue as time processing.

After few previous and older studies in this field [15], the measurement of CFF is a newly used technique in the context of time experience in psychiatric disorders that correlates how we perceive an object and process this in time intervals. Thus, the organism constitutes time units and relations for itself. Since CFF differ concerning age, animal species (dog vs. fly vs. human), personality, specific psychological moments (such as the so-called “slow motion moment”), CFF parameters seem to be exploratory interesting as correlates for possibly changed time perception in patients with mental diseases, although other cognitive mechanisms and influences as masking, persistence or attention have also to be taken into account. The CFF is the frequency at which successive flashes of light are perceived as a continuous light signal. The higher the CFF, the faster we can detect changes in our environment: for example, a fly whose CFF is around 120 Hz can perceive human movement as slowing down considerably; in humans, the CFF lies between 20 and 90 Hz and is dependent on neuronal but also psychological variables, in addition to physical parameters such as light intensity. Therefore, it can be concluded that by measuring the CFF in psychiatric patients, an indication of underlying impairment and pathophysiological correlate of an altered time perception can be obtained. With this research approach combined with neurobiological methods, it may be possible to better attempt to clarify exactly how altered time experience is present in the mentally ill and how relevant it is to the pathophysiology of these disorders.

The aim of this pilot study was therefore to investigate on several levels using simple tasks whether time experience as subjective time feeling and objective time perception are altered in the group of patients with major depression and schizophrenia compared to healthy subjects, and whether they are related to the respective psychopathological symptoms and to what extent these changes are related to the LDAEP and the CFF.

## Methods

### Study population

A group of 34 patients with unipolar depressive disorder (ICD-10: F32.X and F33.X; mean age: 32.4 ± 9.8 years) and a group of 31 patients with schizophrenia (ICD-10: F20.X; mean age: 35.1 ± 10.7 years; see Table 1) were tested. Included patients were at least 18 years old, agreed to participate in the study by signing the informed consent form and spoke the German language. Patients with a comorbid severe neuropsychiatric disorder or addiction (e.g., alcohol and illicit drugs), severe physical diseases with possible cerebral effects (e.g., Cushing’s disease, hepatic encephalopathy, and oncological diseases), the presence of primarily organic brain diseases (e.g., Alzheimer’s dementia and Parkinson’s disease) and the existence of a color vision disorder (ICD-10: H53.5) were excluded. The presence of hearing loss, assessed in the context of the study by a hearing test, also led to exclusion.

**Table 1.** Sociodemographic and clinical psychometric characteristics.

	Patients with schizophrenia *N* = 31	Patients with depression *N* = 34	Healthy controls *N* = 33
Age (years)	35.06 ± 10.08	32.4 ± 9.8	32.85 ± 14.31
Gender	9 Women	13 Women	17 Women
	22 Men	21 Men	16 Men
PSP	57.87 ± 8.70	63.8 ± 5.0	97.27 ± 1.13
CGI	4.74 ± 0.82	4.82 ± 0.67	–
PANSS pos.	17.61 ± 6.01	–	–
PANSS neg.	18.68 ± 6.42	–	–
PANSS ALP	37.23 ± 5.74	–	–
HAM-D	–	25.9 ± 5.3	–
BDI	–	30.1 ± 9.2	–
Age at onset of illness			–
	23.73 ± 5.87	25.0 ± 10.8	

Abbreviations: BDI, beck-depression-inventory; CGI, clinical global index; HAM-D, Hamilton-depression-scale; PANSS, positive- and negative-syndrome scale; PSP, personal and social performance scale.

Existing psychopharmacotherapy was not an exclusion criterion. Antipsychotics were converted into chlorpromazine equivalents so that they could be correlated with the biological and clinical variables. With regard to antidepressants and other drugs, patient subgroups with monotherapy or simple or complex polypharmacy were formed and then statistically compared with each other. All patients were recruited in the Department of Psychiatry of the LWL University Hospital of Ruhr University Bochum during their inpatient treatment. All patients with depression were on antidepressant medication, mainly selective serotonin reuptake inhibitors. All patients with schizophrenia were treated mainly with atypical neuroleptics. Occasionally, the patients received on-demand medication with a low-potency neuroleptic or benzodiazepine.

The control group consisted of 33 healthy volunteers (mean age: 32.8 ± 14.3 years; see Table 1). Healthy subjects were recruited by means of flyers from the authors’ circle of acquaintances, the clinic and a large existing subject file. Prerequisites for study participation were a minimum age of 18 years, written consent to participate and the presence of sufficient German language skills. Exclusion criteria for the healthy subjects were the presence of severe somatic and mental illnesses, existing medication and a positive family history of a mental disorder.

The study was approved by the ethics committee of the Medical Faculty of Ruhr University Bochum (No. 18-6531-BR). Consent to participate in the study was given in accordance with the Helsinki and ICH-GCP declarations.

### Psychometrics

Depressive symptoms were assessed using the Hamilton Depression Scale (HAM-D [16]) and the Beck Depression Inventory (BDI [17]). The Positive and Negative Syndrome Scale (PANSS [18]) was used to assess schizophrenic symptoms. The psychosocial functioning level of patients with schizophrenia was assessed using the German version of the Personal and Social Performance (PSP) scale [19] and the general severity of illness was assessed using the Clinical Global Impression (CGI) scale [20]. In addition, all three groups were given a detailed questionnaire on the sociodemographics and clinical data.

### Time questionnaire

The time questionnaire was used to record the experience of time from the inner perspective (“time feeling”) of the test subjects. This was designed independently, based on the “time and events” section [21, pp. 22–26] of the “Examination of Anomalous World Experience” (EAWE), a detailed interview that captures personal experiences of various aspects of the experienced environment. The EAWE interview is divided into six sections: (a) space and objects; (b) time and events; (c) other persons; (d) language; (e) atmosphere, and (f) existential orientation. Section 2 (“time and events”), in turn, comprises six items that ask whether and to what extent the passing of time is perceived in a changed way. This includes, among other things, changes in the speed and interruptions of the flow of time, differences in internally and externally perceived time and a disturbed relationship to the future and past. The newly designed time questionnaire for self-rating, based on the EAWE scale and used here, contains 20 items with first-person statements on the subjective feeling of time that are oriented toward the categories of a changed experience of time on the EAWE scale described above. In addition, some statements on how the test subjects deal with their own lifetime were added. The statements were evaluated using a visual analogue scale ranging from “not at all true” to “completely true” (0–10). The 20 items of the questionnaire were divided into three categories (time knowledge, seven items; time feeling, seven items; and time handling, six items) based on the time dimensions of Jaspers [22]. For evaluation, both the combined categories in the form of an overall value and each category separately were used. The total values were divided by the number of individual items to standardize the values. The higher these values are, the more problems the test person has overall with time experience or with the subdimensions mentioned.

### PC-based time measurement

To examine the ability to estimate time (task one) and to produce time intervals (second task), in order to assess time perception, a computer-controlled test was carried out (programmed by Arthur Berns, Göttingen). This is an adaptation of the “Chronotest” by Bschor et al. [23].

In the time estimation task, subjects were presented with a light bulb on the computer screen that lit up for a specific time interval predetermined by the investigator. The task was to estimate the duration of this time interval in seconds and enter it in a field provided on the screen. To produce time intervals, the subjects were asked to light up a light bulb for a certain number of seconds by pressing the computer keyboard. For both tasks, six runs each were performed but only runs 2–6 were recorded and analyzed. The data from the first run were discarded in order to avoid habituation effects. The duration of the individual runs was determined before the survey began and was the same for all tests. The duration of the runs in verbal time estimation was 5, 1, 13, 2, and 32 s. For time production, durations were set at 4, 1, 27, 2, and 14 s.

### Critical flicker-fusion frequency

The basic principle of CFF is to measure the frequency of successive light signals. The faster the frequency, the less the signals can be distinguished from each other and are therefore perceived as one continuous signal. With the help of a CFF device, it is possible to measure the frequency at which the test persons perceive the signals as continuous. Measurement of CFF was carried out using a HEPAto-norm analyzer. This was determined by performing two series of measurements (“UP” and “DOWN”) with 10 passes each. The HEPAto-norm analyzer consists of a pair of headband goggles, a handheld control unit and a stop button. The headband goggles were held in front of the subject’s eyes and generated a light-emitting diode (LED) light spot at a frequency of 60 Hz. The frequency was then continuously reduced down to 25 Hz. The subject indicated when the light signal was no longer perceived as continuous by pressing the stop button. The frequency at which the test person was able to distinguish individual light signals for the first time was recorded and listed in the “DOWN” measurement series. To generate the “UP” series of measurements, the frequency of the light signals was continuously increased from 25 to 60 Hz. In this case, subjects pressed the stop button when they perceived the light signal to be constant for the first time (at CFF). The measurement took place in a room darkened by curtains.

### Loudness dependence of auditory evoked potentials

Subjects sat in a comfortable armchair in an electrically shielded and sound-attenuated room. They were instructed to avoid movements and blinking during the entire testing procedure. Auditory evoked potentials were recorded with 32 passive nonpolarizable Ag–AgCl electrodes mounted on an elastic cap in accordance with the 10–20 system of the BrainAmp MR Amplifier and BrainVision Recorder software (Version 1.20.001; Brain Products GmbH, Gilching, Germany) The electrode configuration contained 29 electroencephalography (EEG) channels, one ground, one reference (placed at FCz), and one electrooculography (EOG) electrode. We controlled for ocular artifacts by means of the EOG electrode, which was located 1 cm below the left outer canthus. Impedances were kept at 10 kΩ or below. EEG was filtered using a bandpass filter of 0.531–70 Hz and data were collected at a sampling rate of 250 Hz. A 5–10-min resting EEG was recorded with eyes closed to exclude the presence of any significant EEG abnormalities, such as epileptiform discharges or diffuse EEG slowing. Auditory stimuli were presented binaurally via earphones (Sony Stereo Headphones MDR-1A, Sony Corporation) and using the Presentationsoftware (Neurobehavioral Systems, Inc., Version 14.9; Berkeley, CA, www.neurobs.com). Pure sinus tones (1000 Hz, 40 ms, 10 ms r/f, ISI 1841–2239 ms, mean 2046 ms) of five different intensities (60, 70, 80, 90, and 100 dB sound pressure level) were presented in a pseudorandomized order. A total of 350 sweeps, 70 per intensity, with an epoch length of 800 ms were evaluated. Data analysis was carried out using the BrainVision Analyzer 2.0 (Version 2.2.07383; Brain Products GmbH, Gilching, Germany). In detail, rereferencing to the average of all electrodes was conducted and a notch filter and high- and low-pass filters were applied (low-pass filter 0.5 Hz; high-pass filter 20 Hz). The first response to each of the five intensities was also excluded to reduce short-term habituation effects. After segmentation into the five loudness levels, the epoch 350 ms before stimulus onset was used for baseline correction. Epochs with excessive eye or body movements (±100 μV) in any of the 32 channels were automatically rejected. For each subject, the remaining sweeps were shortened to 300-ms epochs and averaged separately for the five intensity levels. At least 30 artifact-free sweeps in any of the intensities were required with respect to the auditory evoked N1/P2 potential. The N1 amplitude was regarded as the nadir between 50 and 150 ms after the stimulus and P2 as the peak between 100 and 250 ms post-stimulus. The N1/P2 amplitude was then calculated as the difference of peak amplitudes between N1 and P2. The LDAEP of the scalp data (Cz) was calculated as a median exponential slope of the amplitudes of the single loudness levels.

### Statistical analysis

Statistical analyses of the data were performed using IBM SPSS Statistics for Windows, Version 26.0 (IBM Corp., Armonk, NY). Descriptive statistics are given as means, standard deviations, and ranges. Statistical analyses were performed using the appropriate parametric or nonparametric tests (*t*-test, Mann–Whitney *U* test, analysis of variance and Pearson or Spearman correlation coefficients). Statistical significance was taken as *p* < 0.05, with *p* < 0.10 indicating a statistical tendency.

## Results

### Sociodemographic and psychometric findings

The sociodemographic and psychometric findings of the two patient groups and the healthy controls are summarized in Table 1. The patients were clearly impaired according to the nosology-specific psychopathology (BDI, HAM-D, and PANSS) and showed obvious to marked difficulties and limitations in one or more psychosocial functioning domains (PSP).

### Time questionnaire (“time feeling”)

**Figure 1.**
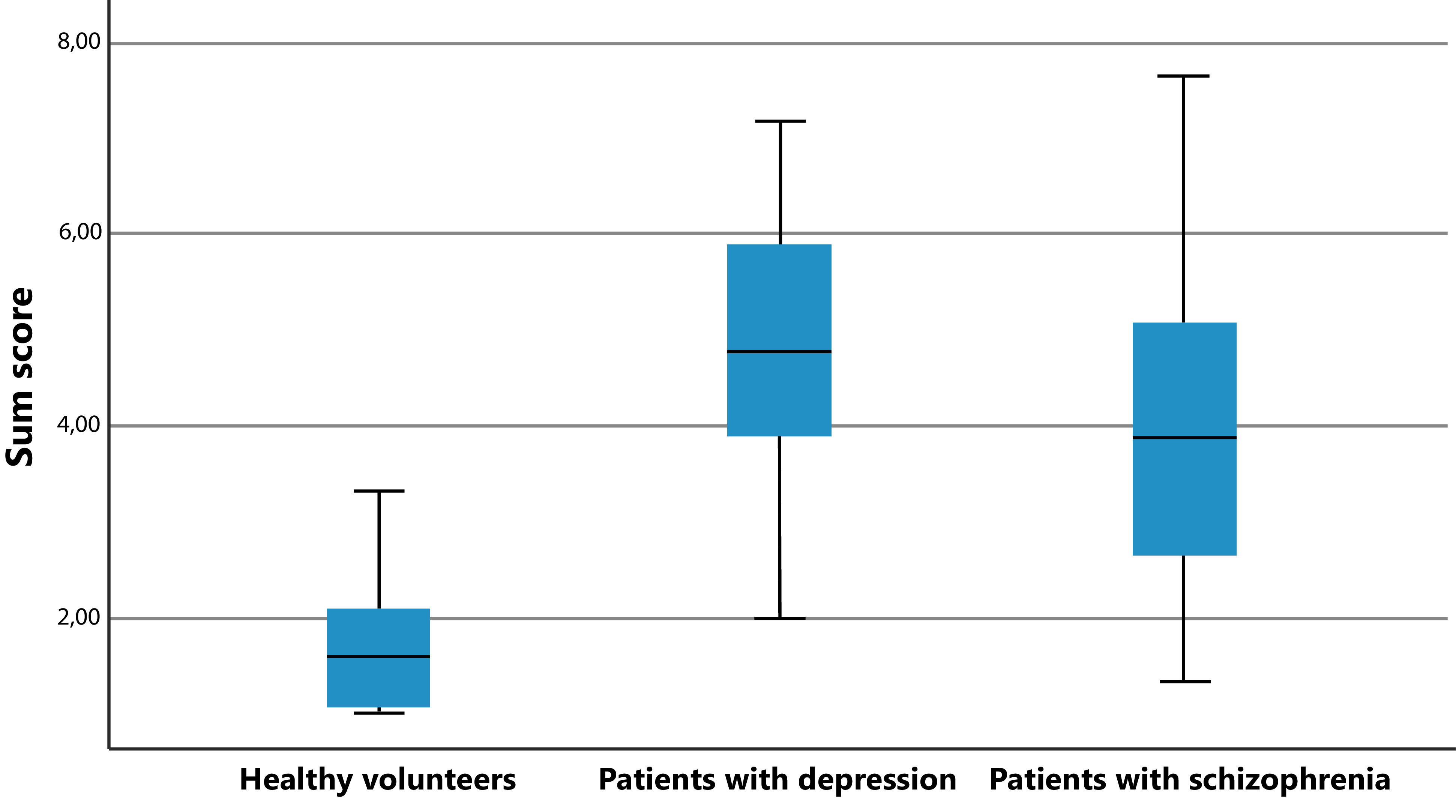
Time questionnaire (sum score of all three groups).

In the time questionnaire, it was found that both the schizophrenic and depressive patients scored significantly higher than the healthy subjects. This applies to the values averaged over the entire questionnaire as well as to the individual consideration of the three categories of time knowledge, time feeling, and time handling (Table 2 and Figure 1).

**Table 2.** Time questionnaire (group differences).

	Schizophrenic patients	Depressive patients	Healthy controls	ANOVA
Time knowledge	3.94 ± 1.73	4.42 ± 1.47	1.78 ± 0.97	*F*_2/97_ = 32.6, *p* < 0.001 Eta^2^ = 0.41
Time feeling	3.39 ± 1.52	4.07 ± 1.60	1.86 ± 0.88	*F*_2/97_ = 22.6 *p* < 0.001 Eta^2^ = 0.32
Time handling	4.20 ± 2.09	6.06 ± 1.77	1.52 ± 0.60	*F*_2/92_ = 67.5 *p* < 0.001 Eta^2^ = 0.59
Sum	3.78 ± 1.47	4.85 ± 1.36	1.74 ± 0.75	*F*_2/97_ = 55.6 *p* < 0.001 Eta^2^ = 0.52

No significant differences were found between the depressive and schizophrenic patients in the post hoc test for the dimension of time knowledge but a statistical tendency was found for time feeling (*p* = 0.08). In contrast, significant differences were found between the two patient groups in time handling (*p* < 0.001) and the sum score (*p* = 0.006), with stronger time experience disorders in the depressed patients.

### PC-based time measurement and CFF

Concerning time perception, a systematic group difference in time estimation was shown as a statistical tendency, which was explained by a significant difference post hoc between the healthy subjects and the schizophrenic patients (*p* = 0.03). However, this was not detectable in the production of time intervals (Table 3).

**Table 3.** PC based time measurement and CFF.

	Schizophrenia	Depression	Healthy	ANOVA
Differences time estimate	0.46 ± 4.26	−0.66 ± 2.95	−1.44 ± 1.88	*F*_2/97_ = 2.95 *p* = 0.057 Eta^2^ = 0.06
Differences time production	0.21 ± 3.19	−0.07 ± 2.33	−0.45 ± 2.40	n.s.
Measurement series UP	36.95 ± 4.26	36.65 ± 3.39	37.07 ± 2.95	n.s.
Measurement series DOWN	39.72 ± 3.79	40.96 ± 2.53	40.92 ± 2.83	*F*_2/97_ = 3.71 *p* = 0.03 Eta^2^ = 0.08

There were no systematic differences in the measured CFF with regard to the UP condition between the studied groups. For the DOWN condition (Table 3) there was a significant difference with a post hoc effect between the two patient groups showing lower CFF for the patients with schizophrenia (*p* = 0.02).

### Loudness dependence of auditory evoked potentials

When comparing the groups, there were no systematic differences between schizophrenics, depressives and healthy persons, either in the analysis of variance or in the post hoc *t*-tests.

### Correlations

No significant influences of age, gender, education, medication, or other sociodemographic variables were found on the time parameters examined here.

There were no significant correlations between the time measures and the LDAEP in the healthy subjects or the depressed patients. However, in the schizophrenic patients it was found that the LDAEP correlated negatively to Cz, Fz, and Pz with the categories of time feeling, time handling, and sum score of the time questionnaire (Table 4). A stronger change in the subjective side of time experience was associated with a weaker LDAEP and thus increased serotonergic neurotransmission. No correlation was found for the time knowledge category of this scale.

**Table 4.** Relationship of LDAEP (recorded on the three central electrodes) and time questionnaire in patients with schizophrenia.

Electrodes	Time feeling	Time handling	Total score
Cz	*r* = −0.308	*r* = −0.401*	*r* = −0.327
	*p* = 0.092	*p* = 0.025	*p* = 0.072
Fz	*r* = −0.421*	*r* = −0.330	*r* = −0.387*
	*p* = 0.018	*p* = 0.070	*p* = 0.031
Pz	*r* = −0.378*	*r* = −0.187	*r* = −0.250
	*p* = 0.036	*p* = 0.314	*p* = 0.176

p<0.05.

Concerning the severity of depressive and schizophrenic psychopathology, correlations were found mainly with the dimensions of the time questionnaire. Time knowledge, time handling, and sum score (but not time feeling) correlated positively with the PSP scale and the BDI (from *r* = 0.36, *p* < 0.05 to *r* = 0.65, *p* < 0.001), but not with the HAM-D, in depressed patients. Time handling and sum score also correlated with the PANSS-negative and PANSS-general in the schizophrenic patients (from *r* = 0.34, *p* < 0.05 to *r* = 0.53, *p* < 0.001). No other significant correlations were found.

## Discussion

In this exploratory study of time experience and possible underlying neurobiological parameters in patients with schizophrenia and depression, significant changes in subjective time feeling were found in both depressed and schizophrenic patients compared to healthy controls. Here, both patient groups showed poorer scores; overall, the depressive patients showed stronger changes in this respect compared to the schizophrenic patients. In the depressed patients, these changes did not correlate with the objective assessment of depressive symptoms and their extent by means of the HAM-D but they did correlate with the self-assessment of these symptoms using the BDI. Interestingly, in the schizophrenic patients time feeling was related to the negative and general psychopathological symptoms and their severity. The schizophrenic patients differed mostly with regard to objective time perception tasks in terms of time estimation, but not with time production. For the CFF (the DOWN condition) there were slightly lower values in the schizophrenic patients, but this was not correlated with time experience or psychopathology. With regard to the serotonergic system (LDAEP), no group differences were found but in the patients with schizophrenia there were significant negative correlations with time feeling, time handling, and the sum score of the time questionnaire, as seen in the pathologically increased serotonergic neurotransmission with disturbed subjective time experience.

It should be mentioned that in this study, time feeling seems to be more clearly disturbed in the two patient groups, including the correlation with the neurobiological variables, while concerning time perception it was a bit differentially found for depression and schizophrenia. This corresponds to previous experience in the literature [1–3,24] that changes in the basal temporal processing and time perception compared to time feeling are less pronounced overall in the classic psychiatric diseases than in organic brain disorders such as dementia, Parkinson’s disease, and so forth. The finding that only patients with schizophrenia showed conspicuous values in time estimation and CFF, while depressive patients were more similar to healthy people, not only fits with the current state of the literature but also with the idea of schizophrenia as a brain development disorder with microfunctional brain changes and correspondingly stronger imaging findings [5]. While concerning time production in the time perception tasks could only seen a similar tendency, patients with depression estimate time intervals shorter comparable with the healthy volunteers as the time duration was given in advance. In contrast, patients with schizophrenia estimate these intervals longer interestingly. This could be possibly interpreted within the context of slowed neuropsychological processes in this disease.

Furthermore, it is interesting that depressive patients have significantly stronger disturbances in time feeling compared to healthy and schizophrenic patients. It is also fitting that this was found to be related only to the assessment of time feeling, but not of time perception in patients with depression. This is in line with many findings in studies with depressive patients: that the subjective suffering with time across the construct “major depression” (from strongly neurotic/environmental to endogenous neurobiological) is felt and reported more strongly by these patients [25] than can be objectified, whereas patients with schizophrenia generally perceive their suffering less and, if they do, suffer more for themselves subjectively and less expressively directed outwards [26].

In this context, the correlation between the values of the time questionnaire regarding time feeling and the negative schizophrenic symptoms seems to be significant because it expresses that the subjective experience of time and temporal relationships is disturbed in schizophrenic patients as an essential feature of daily mental activities, especially in the context of negative symptoms such as apathy, anhedonia, abulia, or alogia. As the influence of depressive symptoms could be excluded here, one could speculate that these main symptoms, which are particularly characteristic of schizophrenia (Bleuler), are possibly also responsible for the fact that patients feel altered in their time feeling (e.g., in the sense of slowing down or fragmentation) [27,28].

With regard to the psychophysiological measurement method of CFF, the results were manageable and only in the DOWN condition was there a small significant effect in the schizophrenic patients. As this was the first application of CFF in psychiatric patients in regard to time experience, the results cannot be considered adequate. However, as there were also no clear relationships to time feling and time perception for the parameters of the CFF, it must be concluded that these are possibly only insufficiently suitable for mapping changed time perception in depressive and schizophrenic patients. Thus, no data are available for CFF and time experience in mental illness so far. There are indications that there is a connection with nonspecific cognitive function losses [29] and CFF also plays a indirect role in the context of learning in motion processing [30]. However, it is not sufficiently known which specific cognitive functions are mapped by CFF. The assumption that CFFs map a biological function of time perception therefore could not be confirmed in this study. It has also to be stated out that CFF measures are related to many other biological and functional parameters across medicine, so that the question remains whether CFF is suitable as objective correlate of any time experience disturbances.

No group differences were found in the LDAEP as an indicator for the serotonergic system, which is surprising in view of previous findings [13,31] or for the known changes in serotonergic neurotransmission for depression and schizophrenia. The effects of medication or other severe psychopathological findings were possibly responsible for this. However, the correlation between the LDAEP and time feeling in the schizophrenic patients is interesting. Why this was not found in depression, a “classic” serotonin disorder, is difficult to explain. The depth of the disturbance of the time experience in schizophrenic patients may again be responsible here, which presumably has a stronger neurobiological foundation and possibly also causes a stronger variance. In any case, the correlative relationship points in the expected direction because a pathologically increased serotonergic neurotransmission is consistently assumed in schizophrenic disorders [6], and this seems to be related to the altered time feeling in schizophrenic patients. Because this is also associated with increased values of negative symptomatology in particular, a possible pathophysiological circle seems to close here, as an altered LDAEP in the sense of increased serotonergic neurotransmission was found particularly in connection with negative schizophrenic symptomatology in these patients [13]: that is, changes in time feeling seem to be an expression of negative symptoms in schizophrenia, the background to which could be changes in the synaptically released serotonin. In this respect, evidence was found for the first time that aspects of time experience and their pathological changes in psychiatric diseases such as schizophrenia are related to the activity of the serotonin system, as measured with the LDAEP.

### Limitations

A limiting factor in this study was certainly the psychopharmacotherapy of the two patient groups. Secondly, only the illness specific psychometric scales were performed in each disorder. Since participants performed time estimation and production trials in identical orders, it can not be excluded that this has had specific effects on, for example, duration judgements. In addition, the group size was possibly not quite sufficient for some aspects of the study, such as the time measurement program and further instrumental diagnostics. In future studies, more meaningful results could be obtained by increasing the number of cases. The time questionnaire was self-compiled, based on international phenomenological templates, not performed within an interview, and had not been validated in a previous study. However, the clear differences in the two patient groups compared to the healthy subjects showed that substantial and valid results could be achieved. It is especially interesting that time feeling determined by the time questionnaire produced particularly clear results in this study. In the sense of an orienting pilot study, no Bonferroni correction was carried out. It is also possible that a stricter definition of the inclusion and exclusion criteria could measure greater effects. For example, the illness phase of the patients was not taken into account. However, the time experience in a psychotic episode for schizophrenic patients could differ, for example, from the time experience at the beginning of the remission phase. To sum up, the most important limitations of this study are lack of precise matching, lack of identical psychometric evaluation, self-rating instead of interview assessment, low trial number, lack of control for order of presentation and carry over effects and the exploratory nature of the experiments.

## Conclusions

This explorative pilot study conducted here aimed to gain an overview of different dimensions of time experience in patients with schizophrenia or depression with the help of heterogeneous methodology. In necessary confirmatory subsequent studies, there is now the possibility of looking at the individual aspects of the experience of time in more detail. Among other things, time feeling could be examined more closely in a more extensive self-assessment questionnaire. The correlations already found in this study could thus be strengthened and possibly provide a basis for taking time experience into account as a parameter in the psychopathological assessment of findings and also in the subsequent diagnosis of psychiatric illnesses to a greater extent than before. More comprehensive understanding of the experience of time in different psychiatric disorders such as depression and schizophrenia should lead to a better understanding of these and other subgroups as the experience of time is an essential dimension of the psyché and its relationship to the world. It is surprising that the attempts of tradition have not led to this [1,22] or established this as an important psychopathological dimension in everyday psychiatric work, although today there is a justified prospect of underpinning such a category accordingly and also neurobiologically with findings from modern methods, ranging from EEG to high-resolution magnetic resonance imaging.

## Data Availability

Further data are available on request.

## References

[1] Juckel G, Steinfath H, Mavrogiorgou P. Disturbances of time experience in mental disorders. Nervenarzt. 2022;93:68–76. doi:10.1007/s00115-020-01047-z. 33403445

[2] Thönes S, Oberfeld D. Time perception in depression: a meta-analysis. J Affect Disord. 2015;175:359–72. doi:10.1016/j.jad.2014.12.057. 25665496

[3] Thönes S, Oberfeld D. Meta-analysis of time perception and temporal processing in schizophrenia: differential effects on precision and accuracy. Clin Psychol Rev. 2017;54:44–64. doi:10.1016/j.cpr.2017.03.007. 28391027

[4] Jacobi F, Höfler M, Strehle J, Mack S, Gerschler A, Scholl L, et al. Mental disorders in the general population: study on the health of adults in Germany and the additional module mental health (DEGS1-MH). Nervenarzt. 2014;85:77–87. doi:10.1007/s00115-013-3961-y. 24441882

[5] Juckel G. Serotonin und akustisch evozierte Potentiale: Auf der Suche nach einem verlässlichen Indikator für das zentrale 5-HT-system. Monographien aus dem Gesamtgebiete der Psychiatrie. Vol. 109. Darmstadt: Steinkopff; 2005.

[6] Müller CP, Cunningham KA. Handbook of the behavioral neurobiology of serotonin. 2nd ed. Oxford: Academic Press; 2020.

[7] von Gebsattel VE. Zeitbezogenes Zwangsdenken in der Melancholie: Versuche einer konstruktiven genetischen Betrachtung der Melancholiesymptome. Nervenarzt. 1928;1:275–87.

[8] Ratcliffe M. Varieties of temporal experience in depression. J Med Philos. 2012;37:114–38. doi:10.1093/jmp/jhs010. 22474140

[9] Fischer F. Zeitstruktur und Schizophrenie. Z Ges Neurol Psychiatr. 1929;121:544–74.

[10] Fuchs T. Selbst und Schizophrenie. Dtsch Z Philos. 2012;60:893.

[11] Kupke C. Der Begriff Zeit in der Psychopathologie. Berlin: Parodos Verlag; 2010.

[12] Treisman M. Temporal discrimination and the indifference interval: implications for a model of “the internal clock”. Psychol Monogr. 1963;77:1–31. 10.1037/h0093864 5877542

[13] Juckel G. Serotonin: from sensory processing to schizophrenia using an electrophysiological method. Behav Brain Res. 2015;277:121–4. doi:10.1016/j.bbr.2014.05.042. 24971690

[14] Juckel G, Molnár M, Hegerl U, Csépe V, Karmos G. Auditory-evoked potentials as indicator of brain serotonergic activity - first evidence in behaving cats. Biol Psychiatry. 1997;41(12):1181–95. doi:10.1016/s0006-3223(96)00240-5. 9171909

[15] Slaghuis WL, Bishop AM. Luminance flicker sensitivity in positive- and negative-symptom schizophrenia. Exp Brain Res. 2001;138(1):88–99. doi:10.1007/s002210100683. 11374087

[16] Hamilton M. Development of a rating scale for primary depressive illness. Br J Soc Clin Psychol. 1967;6:278–96. 6080235 10.1111/j.2044-8260.1967.tb00530.x

[17] Beck A, Ward C, Mendelson M, Mock J, Erbaugh J. An inventory for measuring depression. Arch Gen Psychiatry. 1961;4:561–71. doi:10.1001/archpsyc.1961.01710120031004. 13688369

[18] Kay SR, Fiszbein A, Opler LA. The positive and negative syndrome scale (PANSS) for schizophrenia. Schizophr Bull. 1987;13:261–26. doi:10.1093/schbul/13.2.261. 3616518

[19] Juckel G, Schaub D, Fuchs N, Naumann U, Uhl I, Witthaus H, et al. Validation of the personal and social performance (PSP) scale in a German sample of acutely ill patients with schizophrenia. Schizophr Res. 2008;104:287–93. doi:10.1016/j.schres.2008.04.037. 18595665

[20] Guy W. ECDEU assessment manual for psychopharmacology. Rockville, MD: U.S. Department of Health, Education, and Welfare; 1976.

[21] Sass L, Pienkos E, Skodlar B, Stanghellini G, Fuchs T, Parnas J, et al. EAWE: examination of anomalous world experience. Psychopathology. 2017;50:10–54. doi:10.1159/000454928. 28268224

[22] Jaspers K. Allgemeine Psychopathologie. Ein Leitfaden für Studierende, Ärzte und Psychologen. 4th ed. Berlin: Springer; 1973.

[23] Bschor T, Ising M, Bauer M, Lewitzka U, Skerstupeit M, Müller-Oerlinghausen B, et al. Time experience and time judgment in major depression, mania and healthy subjects. A controlled study of 93 subjects. Acta Psychiatr Scand. 2004;109:222–9. doi:10.1046/j.0001-690x.2003.00244.x. 14984395

[24] Stanghellini G, Ballerini M, Presenza S, Mancini M, Raballo A, Blasi S, et al. Psychopathology of lived time: abnormal time experience in persons with schizophrenia. Schizophr Bull. 2016;42:45–55. doi:10.1093/schbul/sbv052.sbv052. 25943123 PMC4681541

[25] Mavrogiorgou P, Haller K, Juckel G. Death anxiety and attitude to death in patients with schizophrenia and depression. Psychiatry Res. 2020;290:113148. doi:10.1016/j.psychres.2020.113148. 32497968

[26] Juckel G, Kircher T. Schizophrenie. In: Neurobiologie und Psychotherapie: Integration und praktische Anwendung bei psychischen Störungen. Stuttgart: Schattauer; 2013.

[27] Martin B, Franck N, Cermolacce M, Coull JT, Giersch A. Minimal self and timing disorders in schizophrenia: a case report. Front Hum Neurosci. 2018;12:132. doi:10.3389/fnhum.2018.00132. 29686612 PMC5900747

[28] Vogel DHV, Beeker T, Haidl T, Kupke C, Heinze M, Vogeley K. Disturbed time experience during and after psychosis. Schizophr Res Cogn. 2019;17:100136. doi:10.1016/j.scog.2019.100136. 31193856 PMC6543123

[29] Guzel A, Gunbey E, Koksal N. The performance of critical flicker frequency on determining of neurocognitive function loss in severe obstructive sleep apnea syndrome. J Sleep Res. 2017;26:651–6. doi:10.1111/jsr.12531. 28382650

[30] Seitz AR, Nanez Sr JE, Holloway SR, Watanabe T. Perceptual learning of motion leads to faster flicker perception. PLoS One. 2006;1(1):e28. doi:10.1371/journal.pone.0000028. 17183655 PMC1762365

[31] Juckel G, Pogarell O, Augustin H, Mulert C, Müller-Siecheneder F, Frodl T. Differential prediction of first clinical response to serotonergic and noradrenergic antidepressants using the loudness dependence of auditory evoked potentials in patients with major depressive disorder. J Clin Psychiatry. 2007;68:1206–12. doi:10.4088/jcp.v68n0806. 17854244

